# Four‐year outcomes from a multiparametric magnetic resonance imaging (MRI)‐based active surveillance programme: PSA dynamics and serial MRI scans allow omission of protocol biopsies

**DOI:** 10.1111/bju.14513

**Published:** 2018-10-09

**Authors:** Kevin Michael Gallagher, Edward Christopher, Andrew James Cameron, Scott Little, Alasdair Innes, Gill Davis, Julian Keanie, Prasad Bollina, Alan McNeill

**Affiliations:** ^1^ Department of Urology Western General Hospital Edinburgh UK; ^2^ College of Medicine and Veterinary Medicine University of Edinburgh Edinburgh UK; ^3^ Department of Radiology Western General Hospital Edinburgh UK

**Keywords:** prostate cancer, active surveillance, MRI scan, prostate biopsy

## Abstract

**Objectives:**

To report outcomes from a multiparametric (mp) magnetic resonance imaging (MRI)‐based active surveillance programme that did not include performing protocol biopsies after the first confirmatory biopsy.

**Patients and Methods:**

All patients diagnosed with Gleason 3 + 3 prostate cancer because of a raised PSA level who underwent mpMRI after diagnosis were included. Patients were recorded in a prospective clinical database and followed up with PSA monitoring and repeat MRI. In patients who remained on active surveillance after the first MRI (with or without confirmatory biopsy), we investigated PSA dynamics for association with subsequent progression. Comparison between first and second MRI scans was undertaken. Outcomes assessed were: progression to radical therapy at first MRI/confirmatory biopsy and progression to radical therapy in those who remained on active surveillance after first MRI.

**Results:**

A total of 211 patients were included, with a median of 4.2 years of follow‐up. The rate of progression to radical therapy was significantly greater at all stages among patients with visible lesions than in those with initially negative MRI (47/125 (37.6%) vs 11/86 (12.8%); odds ratio 4.1 (95% CI 2.0–8.5), *P* < 0.001). Only 1/56 patients (1.8%) with negative initial MRI scans who underwent a confirmatory systematic biopsy had upgrading to Gleason 3 + 4 disease. PSA velocity was significantly associated with subsequent progression in patients with negative initial MRI (area under the curve 0.85 [95% CI 0.75–0.94]; *P* <0.001). Patients with high‐risk visible lesions on first MRI who remained on active surveillance had a high risk of subsequent progression 19/76 (25.0%) vs 9/84 (10.7%) for patients with no visible lesions, despite reassuring targeted and systematic confirmatory biopsies and regardless of PSA dynamics.

**Conclusion:**

Men with low‐risk Gleason 3 + 3 prostate cancer on active surveillance can forgo protocol biopsies in favour of MRI and PSA monitoring with selective re‐biopsy.

**Abbreviations**
mpMRI: multiparametric MRI
AUC: area under the receiver‐operating curve
PI‐RADS: Prostate Imaging Reporting and Data System

## Introduction

It is now accepted that active surveillance is the first‐line treatment option for men with low‐risk prostate cancer 1, 2.

Most active surveillance programmes involve repeat ‘protocol’ or ‘confirmatory’ prostate biopsy. This is performed 1 year after diagnosis and at varying time points subsequently. The reason for this is that, historically, up to 35% of cases were upgraded 3.

In 2014, the National Institute for Health and Care Excellence (NICE) recommended multiparametric MRI (mpMRI) scanning at the time of diagnosis for men on active surveillance in the UK 4. Other aspects of active surveillance (such as the confirmatory biopsy) were not changed. It is now recognized that MRI targeted biopsy in addition to systematic biopsy improves detection of upgrading 5, 6, 7. There is early evidence that using MRI with or without a PSA adjunct to risk‐stratify men for confirmatory biopsy may allow safe omission of confirmatory biopsy. However, these studies lack long‐term follow‐up to confirm safety 8, 9, 10, 11. Prostate biopsy in men on active surveillance is a painful procedure that contributes to reduced active surveillance compliance 12. It is also a morbid and costly procedure 13.

There is limited evidence about the use of serial MRI scans for monitoring in active surveillance. Four small studies with short follow‐up have been published reporting serial MRI findings 14, 15, 16, 17 in men on active surveillance. One recent study, included 124 men with Gleason 3 + 3 cancer 18. That study demonstrated low rates of progression over three years; however, the role of the early confirmatory biopsy was not assessed, the surveillance protocol involved ongoing ‘protocoled’ biopsies and there were only 23 cases of progression. Larger studies with longer follow‐up, not performing ongoing protocol biopsies, are needed. In addition, many studies using MRI in prostate cancer diagnosis are based on trials, or are conducted at world expert centres. These studies use 3‐Tesla MRI, expensive targeting software and strict protocols. We wanted to study the use of 1.5‐Tesla mpMRI in active surveillance in the ‘real‐life’ setting.

Prostate‐specific antigen dynamics have had mixed associations with prostate cancer‐specific mortality in men with initial conservative management 19, but there is little 20 to no research to date on the use of PSA dynamics in helping to ‘select’ men on active surveillance for further investigation.

We hypothesize that protocol biopsies can be safely replaced by selective biopsy and a monitoring programme of repeat MRI scans, PSA testing and DRE.

The aim of this study was to determine outcomes for men in a 1.5‐Tesla mpMRI‐based active surveillance programme, and to determine if PSA dynamics could predict subsequent progression in men who remained on active surveillance after an initial confirmatory biopsy.

## Methods

### Study Design and Patients

We conducted an analysis of a prospectively collected observational clinical database.

All patients were diagnosed with Gleason 3 + 3 prostate cancer (2010–2015) by 10‐core systematic TRUS biopsy, performed because of an elevated PSA level. All patients had PSA <15 ng/mL and ≤cT2a disease. Patients underwent DRE in clinic annually and 3‐monthly PSA tests, with data recorded at each visit in our database.

All patients at our institution were included if they underwent mpMRI after TRUS diagnosis of Gleason 3 + 3 prostate cancer and were on active surveillance. We did not have a protocol regarding timing of the first mpMRI scan. This varied based on the patient's risk (number of positive cores, PSA level, DRE findings), patient preference and waiting lists. All patients were offered a confirmatory biopsy after their first MRI scan, regardless of MRI findings. If patients remained on active surveillance after a confirmatory biopsy the MRI was repeated after 2 years. MRI was repeated sooner if there was a rising PSA, concerning findings on DRE or high‐risk features such as more than two positive cores, or a relatively high PSA level for a given prostate volume. However, we did not use set PSA density or kinetic cut‐offs. We did not have a routine re‐biopsy schedule after the confirmatory biopsy at 1 year.

### PSA Dynamics

We hypothesized that PSA dynamics could be useful in guiding further investigation when assessed *after* the first MRI scan. Therefore, we chose a period of PSA testing from up to 12 months before the first MRI scan until up to 18 months after it to calculate PSA dynamics. Equations used for PSA dynamics were: PSA doubling time (least squares linear regression of the log_2_ of at least five PSA values expressed in years) and PSA velocity (last log_2_ PSA of series divided by the series PSA doubling time). PSA dynamics were assessed for association with progression to radical therapy in patients who remained on active surveillance after first MRI (with or without confirmatory biopsy).

### Magnetic Resonance Imaging

The MRI was performed on 1.5‐Tesla Siemens systems at five different hospitals. All MRI scans included dynamic contrast enhancement (8‐s temporal resolution), anatomical and functional diffusion‐weighted imaging (B‐values: 50, 600 and 1200) with pelvic phased array (no rectal coils used). Primary MRI reports were by mixed specialist and non‐specialist radiologist. However, all scans were reviewed in a multidisciplinary team with the presence of expert uro radiologists. MRI results were scored on a three‐point scale: ‘no lesion’; ‘moderate‐risk lesion’; or ‘high‐risk lesion’. Moderate‐risk lesion was defined as a visible abnormality of uncertain malignant potential. High‐risk lesion was defined as a visible abnormality that was probably malignant.

Repeat MRI scans were all compared to initial imaging by the reporting radiologist. These were then coded by the study investigators as (i) ‘no change/better’ or (ii) ‘worse’. ‘Worse’ was defined as any one of the following: (i) new lesion identified; (ii) increase in size of previous lesion; (iii) a moderate‐risk lesion now a high‐risk lesion; (iv) new or increased diffusion restriction; (v) new or increased dynamic contrast enhancement; (vi) new evidence of extraprostatic extension. ‘Better’ was defined as at least a one‐point reduction in MRI risk group.

### Biopsies

#### Confirmatory Biopsy

Confirmatory biopsy was offered to all men regardless of MRI findings. This was the first biopsy after diagnosis, following the first MRI scan. If there was a visible lesion on MRI, the confirmatory biopsy was a targeted biopsy along with systematic sampling. Targeted TRUS biopsy involved 2–4 cores targeted to the lesion, plus systematic sampling up to a total of 10 cores. Transperineal targeted biopsy was only performed if an anterior lesion was seen and involved a minimum of four cores, targeted to the lesion, plus saturation biopsy of the whole gland (between 24 and 42 cores total).

#### Selective Repeat Biopsy

Following the confirmatory biopsy no further biopsies were protocoled. Selective repeat biopsy was considered on a case‐by‐case basis with no set criteria. Decision‐making was based on PSA monitoring, annual DRE, serial MRI findings and patient preference. Selective repeat biopsy was TRUS, unless an anterior lesion was seen on MRI or suspected (e.g. MRI‐negative patients with rising PSA level and low‐volume disease on TRUS biopsy) in which case transperineal biopsy was performed.

### Follow‐up

Outcomes were assessed by review of electronic patient notes. The outcomes assessed were as follows: (i) rate of progression to radical therapy at confirmatory biopsy; (ii) rate of progression to radical therapy in patients who remained on active surveillance after confirmatory biopsy; and (iii) the overall rate of progression to radical therapy. Radical therapy was defined as curative intent brachytherapy, external beam radiotherapy or radical prostatectomy. Patients who died from other causes or were converted to watchful waiting (PSA monitoring only, and consideration of hormone therapy if applicable) were included in the ‘non‐progression’ category. These patients avoided radical therapy and did not come to harm from prostate cancer. Patients were converted to watchful waiting when it was agreed between patient and clinician that further invasive investigation was no longer of benefit.

### Statistical Analysis

Continuous variables are recorded as mean (95% CI; normally distributed) or median (25th, 75th; skewed or kurtotic) and compared using Student's *t*‐test or the Mann–Whitney *U*‐test, respectively. Categorical variables are displayed as number and percentage, and compared using the chi‐squared test. Survival analysis was performed by Kaplan–Meier plot and groups compared using Cox regression. Significance was accepted at *P* < 0.05. Statistics were analysed using SPSS (SPSS Inc., Chicago, IL, USA) v.21. PSA dynamics were calculated using the ‘LINEST’ least squares regression function in Microsoft Excel v1804.

### Ethics

This project was an audit and was exempt from ethical review at our institution.

## Results

**Figure 1:**
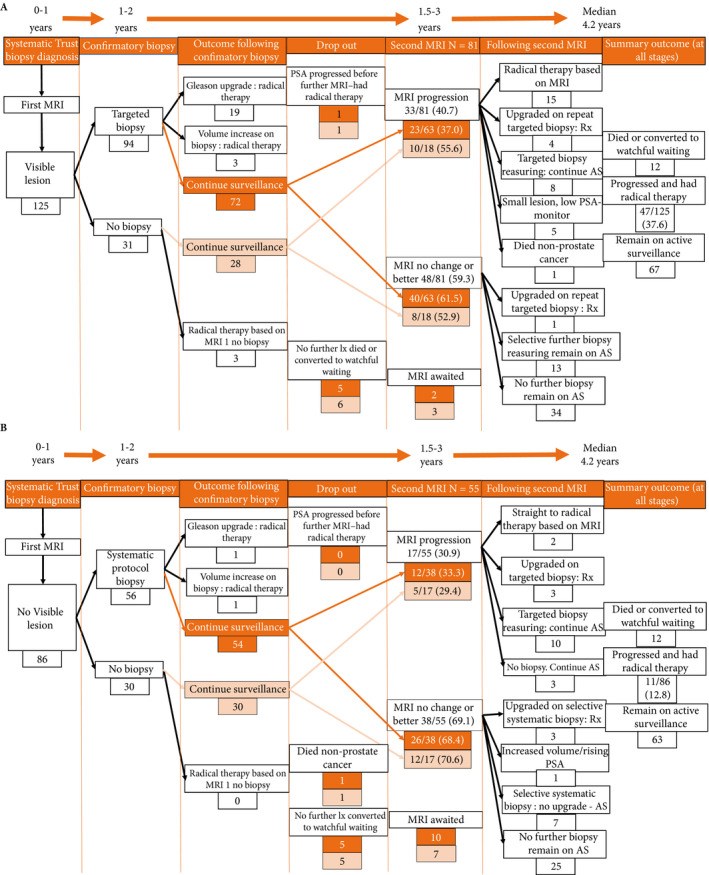
The flow of patients through the study. (**A**) Patients with visible lesions on MRI 1. (**B**) Patients with no visible lesion on MRI 1. All 28 patients with visible lesions who did not have an initial confirmatory biopsy had small lesions with stable PSA and elected for PSA monitoring and repeat MRI before undergoing a biopsy. AS, active surveillance; Rx, treatment.

A total of 401 patients started active surveillance for Gleason 3 + 3 prostate cancer during the period, of whom 211 underwent mpMRI after diagnosis and were included (Table 1). The first mpMRI scan was at a median of 12.1 (7.3–27.5) months after diagnostic TRUS biopsy. Of the 211 patients, 150 (71.2%) underwent a confirmatory biopsy after the first MRI (Table 2); 125/211 patients (59.2%) had a lesion on MRI that was considered targetable, 97 were thought to be at high risk based on MRI, 28 were thought to be at moderate risk, and 86 had no visible lesion (Table 2, Fig. 1). Three patients underwent radical therapy based on first MRI findings alone without confirmatory biopsy. Fifty‐eight patients elected not to have a confirmatory biopsy and to continue PSA monitoring with a view to repeating MRI (Fig. 1). Seven patients were taking 5‐α‐reductase inhibitors (PSA values doubled for reporting) at the time of their first MRI, three with visible lesions and four with no visible lesion.

**Table 1:** Baseline characteristics of patients in the study, with and without progression, defined as requirement for radical therapy

	All	No progression	Progression	Odds ratio	*P*
Number of men, *n* (%)	211	153 (72.5)	58 (27.5)	–	–
Mean age, year	65.3 (64.5,66.1)	65.1 (64.2, 66.1)	65.6 (64.0, 67.2)	–	0.55
Median number of positive cores	1 (1–2.25)	1 (1–2)	2 (1–4)	–	0.001
More than two positive cores	50 (23.7)	25 (16.3)	25 (43.1)	3.88 (1.98–7.60)	<0.001
Median percentage length cores involved	5 (2.5–10)	5 (2.5–10)	5 (2.5–15)	–	0.05
Mean PSA	6.8 (6.2, 7.3)	6.9 (6.1, 7.6)	6.5 (5.7, 7.2)	–	0.89
Median PSA density	0.11 (0.08–0.17)	0.10 (0.08–0.16)	0.12 (0.10, 0.18)	–	0.03
Visible lesion on first MRI, *n* (%)	125 (59.2)	78 (51.0)	47 (81.0)	4.1 (2.0–8.5)	<0.001
Had confirmatory biopsy, *n* (%)	151 (71.6)	105 (68.6)	46 (79.3)	1.8 (0.9–3.6)	0.13

Values are mean (95% CI) or (median 25th, 75th) unless otherwise specified. Reasons for radical therapy are described in the text.

**Table 2:** Confirmatory biopsy findings after first MRI

	All	No visible lesion	High‐risk lesion	Moderate‐risk lesion
Number of men, *n* (%)	211	86 (40.8)	97 (46.0)	28 (13.3)
TRUS, *n*	139	54	68	17
Transperineal, *n*	12	2	7	3
Total had confirmatory biopsy, *n* (%)	150 (71.1)	56 (65.1)	74 (76.3)	20 (71.4)
Any upgrade, *n*/*N* (%)	23/150 (15.3)	1/56 (1.8)	17/74 (23.0)	5/20 (25.0)
Benign, *n*	39	25	10	4
3 + 3	89	30	48	11
3 + 4	21	1	15	5
4 + 3	2	0	2	0
>4 + 3	0	0	0	0
Volume increase on biopsy prompting radical therapy	4	1	3	0

### Overall Progression Rates

**Figure 2:**
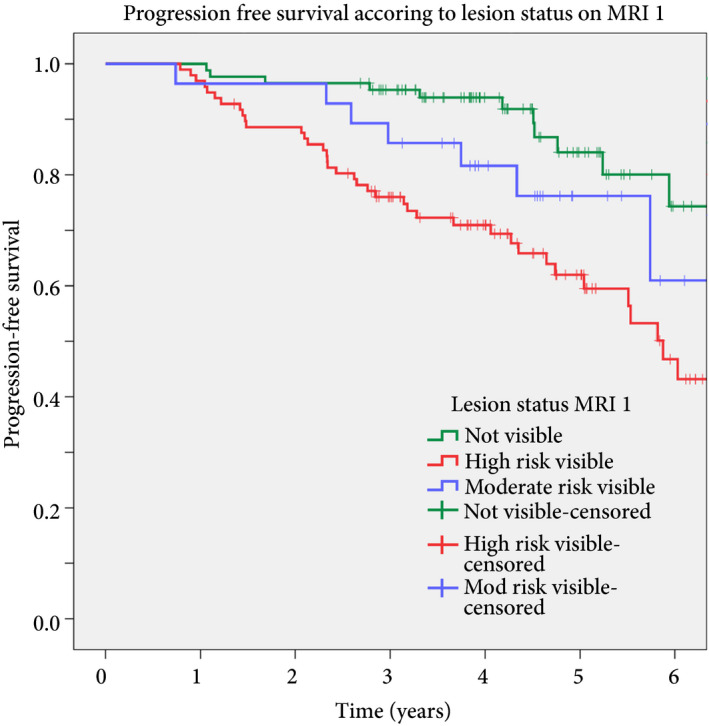
Kaplan–Meier survival analysis categorized by no visible lesion (*n* = 86), moderate‐risk visible lesion (*n* = 28) and high‐risk visible lesion (*n* = 97). Graph truncated at 6 years. Cox regression hazard ratio high‐risk vs no lesion: hazard ratio 3.5 (1.8–6.9), *P* < 0.001; moderate‐risk vs no lesion: hazard ratio 1.99 (0.77–5.14), *P* = 0.15.

With a median of 4.2 (3.4–5.1) years of follow‐up, 58/211 patients (27.5%) had progressed to need radical therapy. Patients with visible lesions on first MRI had significantly higher rates of progression to radical therapy (Table 1, Figs 1A and 2) than patients with no visible lesion on first MRI (Fig. 1B; final progression rate: visible lesion on first MRI: 47/125 (37.6%) vs no visible lesion on first MRI: 11/86 (12.8%), odds ratio 4.1 (95% CI 2.0–8.5); *P* < 0.001). Compared to no visible lesion on first MRI, progression‐free survival was significantly worse in patients with high‐risk visible lesions (hazard ratio 3.5 [95% CI 1.8–6.9]; *P* < 0.001) and trended to worse with moderate‐risk lesions (hazard ratio 1.99 [95% CI 0.77–5.14]; *P* = 0.15 [Fig. 2]).

### Reasons for Radical Therapy

Five patients elected to receive radical therapy based on increased volume Gleason 3 + 3 disease on confirmatory or selective repeat biopsy, usually accompanied by a rising PSA level. Two patients with Gleason 3 + 3 cancer after targeted confirmatory biopsy of a visible lesion at first MRI subsequently had a rising PSA level and elected to undergo radical therapy without further investigation. In all others (51/58), progression to radical therapy was prompted by either upgrading on biopsy, T3 on MRI or progressive high‐risk lesion on MRI. All patients who were converted to watchful waiting were converted before PSA progression.

### Gleason Upgrading on Confirmatory Biopsies after First MRI

The rate of Gleason upgrading was significantly greater in patients with a targetable abnormality on first MRI (22/94 [23.4%] vs 1/56 [1.8%]; odds ratio 16.8 [95% CI 2.20–128.54]; *P* = 0.007 [Table 2]). Patients with ‘high‐risk’ and ‘moderate‐risk’ lesions had similar rates of upgrading on confirmatory biopsy (Table 2). Three patients had upgrading to Gleason 3 + 4 on confirmatory biopsy of a visible lesion but did not choose radical therapy when offered, all required radical therapy after further surveillance.

### Management of Those Still on Active Surveillance after First MRI with or Without Confirmatory Biopsy

In patients who remained on active surveillance after first mpMRI scan with or without confirmatory biopsy (*N* = 184), the rate of subsequent progression to radical therapy in those with visible lesions was 22/100 (22.0%) and 9/84 (10.7%) in patients with no visible lesions initially. For high‐risk visible lesions the progression rate was 19/76 (25.0%) vs 3/24 (12.5%) for moderate‐risk visible lesions.

After first mpMRI (with or without confirmatory biopsy) 81/89 and 55/75 eligible patients with and without visible lesions, respectively, had repeat (second) mpMRI (Fig. 1). The median time between first and second MRI was 29.0 (23.0–34.0) months for patients with no visible lesions and 26.7 (19.5–33.0) months for patients with visible lesions.

### Management after Second MRI

In patients who had a second MRI scan, radiological progression was seen in 33/81 patients (40.7%) with known visible lesions. In patients with no visible lesions on first MRI scan 17/55 patients (30.9%) developed lesions on second MRI scan.

Significant differences in the need for radical therapy at this stage were seen based on the combined findings of the two scans (Table 3). Patients with an unchanged (*n* = 45) or better (*n* = 3) visible lesion had the lowest progression rate (1/48 [2.1%]) vs patients with a new visible lesion (5/17 [29.4%]), or a progressive visible lesion (19/33 [57.6%]) (Table 3). Decision‐making regarding the need for selective repeat biopsy is described in Table 3. Time between second MRI and selective repeat biopsy ranged from 0.2 to 9 months (median 2.4 months) for patients with worsening MRI results and from 0.5 to 17 months (median 2.05 months) for patients with no change on MRI.

**Table 3:** Requirement for radical therapy after second MRI, grouped by comparative findings between first MRI and second MRI

MRI 1	No visible lesion		Visible lesion	
MRI 2	No visible lesion	Developed new lesion	Stable/better lesion	MRI Progression*
*N*	38	17	48†	33
Required radical therapy	4 (10.5)	5 (29.4)	1 (2.1)	19 (57.6)
Straight to radical therapy no biopsy	0	2	0	15
Selective repeat biopsy*	11 (3 upgraded, 8 not)	11 (3 upgraded, 9 not)	14 (1 upgraded, 13 not)	12 (4 upgraded, 8 not)
Reasons for selective repeat biopsy				
New/worsening lesion	0	11/11	0	12/12
DRE changes	3	–	3	–
Rising PSA	6 (3 upgraded – 1 on TP biopsy done as PSA felt out of keeping with TRUS findings)	–	6	–
Persisting high‐risk lesion: concern not adequately sampled first time	–	–	3 (1 upgraded)	–
Patient request	2	–	2	–
Reasons for no biopsy	*N* = 23: no MRI lesion, no other indication to repeat. Continue PSA and MRI monitoring.	*N* = 2: small lesion, low PSA: repeat MRI in 1 year *N* = 1: patient refused, against medical advice *N* = 1: awaiting biopsy	*N* = 34: Stable DRE and PSA, no MRI change, repeat MRI in 1 year	*N* = 2: Awaiting biopsy *N* = 3: Small lesions, low PSA, already been targeted with reassuring findings – await a repeat MRI in 1 year with close PSA monitoring *N* = 1: Died, non‐prostate cancer

Decision‐making in selective repeat biopsy. *Definitions of MRI progression can be found in the methods section. Reasons for radical therapy are described in the text. ^†^Only three men had lesions that were considered ‘better’. All had high‐risk visible lesions initially.

### PSA Dynamics for Prediction of Progression in Patients who Remained on Active Surveillance after Confirmatory Biopsy

**Figure 3:**
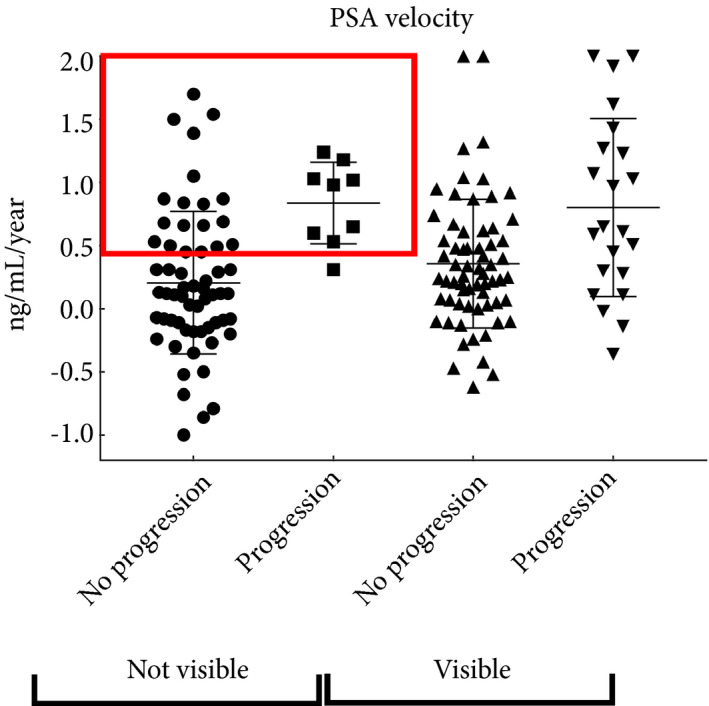
PSA velocity in patients who remained on active surveillance after the first MRI scan (with or without confirmatory biopsy). Progression is defined as requiring radical therapy. PSA velocity values are truncated excluding outliers to allow visualization of the mid‐range values. Red box represents a cut‐off 0.5 ng/mL/year where these patients could be selected for earlier repeat investigation.

When calculated 12–18 months after the first MRI using at least five PSA values, PSA velocity was significantly associated with subsequent requirement for radical therapy in patients with no visible lesions, and PSA doubling time was significant in patients with visible lesions (Table 4). The area under the receiver‐operating curve (AUC) for PSA velocity for prediction of progression in MRI‐negative patients was 0.85 (95% CI 0.75–0.94); for doubling time in MRI‐positive patients, the AUC was 0.65 (95% CI 0.52–0.78). In patients with no visible lesions on first MRI, a cut‐off of 0.5 ng/mL/year in PSA velocity had a sensitivity of 89% (8/9 progressions identified) and a specificity of 75% for progression to radical therapy (Fig. 3). Having more than two positive biopsy cores at baseline was also significantly associated with subsequent progression in patients with visible lesions (Table 4).

**Table 4:** Factors associated with subsequent requirement for radical therapy in men who remained on active surveillance after the first MRI, with or without confirmatory biopsy

Remained on active surveillance after MRI 1 ± confirmatory biopsy									
Lesion status MRI 1	All			Visible			Non‐visible		
	No progression	Progressed	*P*	No progression	Progressed	*P*	No progression	Progressed	*P*
Total	153	31		78	22		75	9	
Median PSA velocity, ng/mL/year	0.19 (−0.10 to 0.54)	0.97 (0.35–1.22)	**0.001**	0.25 (0.04–0.24)	0.63 (0.24–1.3)	0.07	0.12 (−0.16 to 0.51)	0.98 (0.56–1.11)	**0.004**
Median PSA doubling time, years	4.5 (−2.27 to 10.1)	3.3 (2.4–5.8)	0.12	5.7 (2.5–11.1)	3.2 (1.9–5.2)	**0.008**	3.72 (–3.5–9.9)	3.59 (3.1–5.9)	0.95
Mean PSA, ng/mL	6.6 (5.8, 7.5)	6.6 (5.5–7.7)	0.90	7.0 (5.4,8.5)	6.9 (5.4–8.5)	0.91	6.3 (5.8,7.9)	5.8 (4.2,7.4)	0.99
Median PSA density, ng/mL/mL	0.11 (0.84–0.15)	0.12 (0.10–0.16)	0.07	0.11 (0.08–0.15)	0.13 (0.11–0.20)	0.23	0.10 (0.08–0.15)	0.12 (0.07–0.15)	0.48
Mean age, years	64.2 (63.3, 65.1)	65.1 (62.8–67.4)	0.94	65.2 (63.9–66.4)	64.6 (61.5–67.8)	0.71	64.1 (62.7, 65.5)	66.3 (63.5,69.2)	0.87
More than two positive cores, *n* (%)	21 (13.7)	11 (35.5)	**0.001**	13 (16.7)	10 (45.5)	**0.007**	8 (10.7)	1 (11.1)	0.96
Median no. positive cores	1.0 (1.0–2.0)	2.0 (1.0–4.0)	0.28	1.0 (1.0–2.0)	2.5 (1.0–4.0)	0.05	1 (1–2)	1 (1–2)	0.85
Median % length of cores positive, mm	5.0 (2.5–10.0)	5.0 (2.5–14.4)	0.30	5.0 (2.5–10.0)	6.0 (2.5–14.4)	0.40	5.0 (2.5–6.75)	2.5 (2.12–11.9)	0.72

Visible lesion here includes ‘high‐risk’ and ‘moderate‐risk’ visible lesions. Values are mean (95% CI) or median (25th, 75th) unless otherwise specified. Bold values highlight significant variables.

### Patients Who Did Not Have a Confirmatory Biopsy after First MRI

There was no difference in the rate of progression overall at any stage for patients with no visible MRI lesion who had a confirmatory biopsy (7/56 [12.5%]) vs those who did not (4/30 [13.3%]; *P* = 0.87). In patients with visible lesions, of those not requiring treatment based on first MRI, 28/122 (22.9%) elected not to have an initial targeted biopsy because of small or moderate‐risk lesions with low/stable PSA, in favour of MRI and PSA follow‐up. Four of these 28 (14.3%) progressed to radical therapy at last follow‐up, three had progressive lesions on second MRI and one had a rising PSA level and elected to undergo treatment without further investigation (Fig. 1A).

### Were Patients Treated Within the ‘Window of Curability’ if they Remained on Active Surveillance after Stage 1?

In patients who remained on active surveillance after first MRI, (with or without confirmatory biopsy), and went on to later require radical therapy, 25/31 had a PSA level < 10 ng/mL at the time of radical therapy and six had a PSA level >10 ng/mL. Their diagnosis and pre‐radical therapy PSA values were as follows: 7.9–14.1, 11.5–14.3, 14.7–11.1 ng/mL (160 mL prostate volume), 6.7–13.3, 7.2–16.4 and 3.5–10.8 ng/mL. Three of the 31 patients had possible early T3 disease on a second MRI and underwent radiotherapy without further biopsy, all had visible lesions initially.

## Discussion

The aim of the present study was to describe outcomes for men on active surveillance using mpMRI‐, PSA‐ and DRE‐based monitoring with selective re‐biopsy. We showed that routinely performing confirmatory biopsy in men with no visible lesion on MRI has a low diagnostic yield. We then showed that sequential MRI scans with PSA monitoring can identify disease progression regardless of lesion status on first scan. Finally, we showed that PSA dynamics are associated with subsequent progression in men with negative MRI results.

This is the first study, to our knowledge, reporting MRI progression rates in patients with known low‐risk prostate cancer, without protocoled biopsy beyond the first year and with 4 years of follow‐up. It is also the first study, to our knowledge, that assessed PSA dynamics in patients who remained on active surveillance after the first MRI scan/confirmatory biopsy.

Only one out of 56 patients with no visible lesion on first MRI had upgrading at routine confirmatory biopsy. This patient had organ‐confined low‐volume Gleason 3 + 4 cancer at radical prostatectomy. In patients with no visible lesions, repeat MRI identified new lesions in 32%, although only 4/17 needed radical therapy at that stage. Even when a second MRI was still negative, PSA dynamics and DRE selected 11/38 of these patients for repeat systematic biopsy. Of these *selective* repeat biopsies in MRI‐negative patients, 36% had upgrading; thus, when the first MRI is negative, the diagnostic yield of repeat MRI with delayed selective biopsy is significantly higher than performing a routine confirmatory biopsy in all.

The practice of routine systematic confirmatory biopsy for men on active surveillance was based on evidence from the pre‐MRI era that up to 35% of men would be upgraded at repeat biopsy 3. This is no longer the case when MRI risk stratification is taken into account. Other studies have shown that ~10% of men on active surveillance with no visible MRI lesion will be found to have upgrading if routine confirmatory biopsies are performed 8, 21, 22. In the present study, only 1.8% of patients with negative MRI were found to have upgrading on confirmatory biopsy. Our patient populations are similar to previous studies in terms of clinical risk predictors 8, 21, 22; however, in the present study, 35% of patients with no visible lesion elected not to have a routine confirmatory biopsy and to await repeat MRI and PSA monitoring.

Regardless of the confirmatory biopsy findings in MRI‐negative patients, during 4 years of follow‐up, MRI and PSA monitoring with selective re‐biopsy identified 10% requiring radical therapy. This is similar to the rate of radical therapy required in MRI‐negative patients in other studies 5. Furthermore, after the confirmatory biopsy, only 23 of the remaining 84 MRI‐negative patients needed to be subjected to a repeat biopsy to identify these 10% with upgrading. Finally, the rate of progression over the course of 4 years was the same in patients who had a confirmatory biopsy as in those who did not. Thus, we believe the routine, protocoled confirmatory biopsy, could have been safely omitted in patients with negative MRI scans, in favour of further monitoring with selective re‐biopsy.

We accept that since most of our confirmatory biopsies were systematic TRUS biopsies it is possible more upgrading may have been identified in MRI‐negative patients had transperineal template biopsies been undertaken. There is some evidence to suggest this may be the case 23; however, there is no evidence that this is beneficial for long‐term outcomes. We would not recommend more aggressive investigation of MRI‐negative patients with very low‐risk prostate cancer at baseline, given the low rates of progression and safe identification of progression with MRI/PSA monitoring shown in the present study.

In patients with reassuring biopsy of a visible lesion who remained on active surveillance, 41% had some form of MRI progression. Half of these underwent radical therapy (either based on repeat MRI findings alone or following a repeat targeted biopsy). Patients with high‐risk lesions account for the majority of these. In contrast to patients with high‐risk lesions on first MRI, patients with moderate‐risk lesions who had reassuring confirmatory biopsies approached the rate of progression in patients with no visible lesions (12.5% vs 10%). The rate of upgrading at targeted confirmatory biopsy of visible lesions in the present study was lower than that reported elsewhere 5 (23% vs 35%). However, with 4 years’ follow‐up, 37% of patients with visible lesions on first MRI required radical therapy. All but one patient with a visible lesion and subsequent need for radical therapy demonstrated clear MRI progression. This suggests that true cancer progression, rather than inadequate targeting may explain progression in these patients. MRI surveillance with or without selective repeat biopsy was able to identify all patients who progressed before any significant PSA progression. Of 33 patients with MRI progression, 15 were able to progress straight to radical therapy without further biopsy, again reducing the number of biopsies required.

PSA velocity was strongly associated with progression in patients who remained on active surveillance with negative MRI. In patients with negative MRI, PSA velocity should be given weight when selecting for earlier repeat MRI ± biopsy, accepting there is overlap in values between patients with and without progression. PSA dynamics showed less clear association with progression in patients with visible lesions and are unlikely to be clinically useful in this circumstance (Fig. 3). We accept that PSA dynamics may not be predictive of long‐term prostate cancer‐specific mortality in men on active surveillance 19, 24. We believe that PSA velocity can be used specifically to select men on active surveillance with negative MRI for further investigation and is complementary to a holistic clinical assessment in this circumstance.

A limitation of the present study is that our population represents a ‘very‐low‐risk’ active surveillance population. This is because we only included patients who had mpMRI after starting active surveillance. Prior to 2014 many patients had simple MRI scans for local staging and thus could not be included. We also did not have data on those who progressed to radical therapy prior to the first MRI scan. This is in fact true of most similar studies in the field 21, 22, but must be borne in mind when interpreting these studies and ours. It does not detract from results related to follow‐up of patients who remained on active surveillance.

Our clinical service did not use a standardized MRI risk categorization or reporting template (such as Prostate Imaging Reporting and Data System [PI‐RADS] 25 or the PRECISE recommendations 26). MRI risk was assessed at multidisciplinary team review. Visible abnormalities were reported as likely to be malignant or equivocal for malignancy. It is possible that five‐point MRI risk categorization may have better risk‐stratified patients in a larger cohort 11. Kaplan–Meier analysis showed well‐calibrated risk of progression based on the three MRI risk levels that we assigned. We believe there would have been minimal additional prognostic discrimination from a five‐point scale in the present population.

This was a prospective observational study of our normal clinical practice; therefore, we did not record radiological/pathological correlation at radical prostatectomy. We also did not formally double‐report MRI to allow concordance assessment. Validation of the accuracy of MRI reporting is provided by the upgrading at targeted biopsy and radical therapy progression rates in the three lesion risk groups we assigned (Table 2, Fig. 2). We believe this reflects ‘real‐life’ active surveillance and MRI reporting outside of the trial setting. Such data are useful and complementary to controlled trials.

In summary, routine confirmatory biopsy in patients with MRI‐negative, TRUS Gleason 3 + 3 cancer on active surveillance has low diagnostic yield. Subsequent follow‐up of MRI‐negative patients with PSA/MRI/DRE without further protocol biopsies demonstrated low progression rates and timely identification of those in need of radical therapy. Patients with malignant visible lesions are a high‐risk group even after reassuring targeted biopsy. PSA dynamics have limited value in patients with high‐risk MRI lesions. We suggest that patients with high‐risk lesions on first MRI who remain on active surveillance after a targeted biopsy should be offered an early repeat MRI (at 1 year or sooner) regardless of PSA dynamics. Patients with negative MRI, or reassuring biopsy of low‐/moderate‐risk lesions, can defer repeat MRI for 2 years, unless the PSA is rising or DRE changes.

In conclusion, men with low‐risk Gleason 3 + 3 prostate cancer on active surveillance can forgo protocol biopsies in favour of MRI and PSA monitoring with selective repeat biopsy.

## Conflict of Interest

KMG receives research funding from GlaxoSmithKline for a project unrelated to this work. All other authors have nothing to declare.
